# Acute presentation of giant sporadic vestibular schwannoma with massive hemorrhage: A critical review with a case illustration

**DOI:** 10.1016/j.bas.2022.100897

**Published:** 2022-06-06

**Authors:** Nitish Nayak, Anil Kumar Sharma, Surendra Kumar Gupta, Prashant Raj Singh

**Affiliations:** Department of Neurosurgery, All India Institute of Medical Sciences, Raipur, Chhattisgarh, 492099, India

**Keywords:** Giant vestibular schwannoma, Intratumoral hemorrhage, Cerebellopontine angle lesion

## Abstract

**Introduction:**

Hemorrhage in vestibular schwannoma (VS) is a rare but potentially devastating complication, owing to the proximity to the brainstem and small space in the posterior fossa.

**Research question:**

it is a challenge to manage such patients, coming in emergency in comatose state and whether early intervention can reverse the consequence or not.

**Material and methods:**

A 42-year-old male presented in emergency room (ER) with sudden onset of severe headache followed by loss of consciousness, with GCS- 3 (E1V1M1) and mid-dilated fixed pupil. Computer tomography (CT scan) head revealed a large hematoma in the right cerebellopontine angle (CP) with hydrocephalus, requiring urgent CSF diversion (External Ventricular Drain) in the ER. Subsequently microsurgical excision of the tumor was done after few days, once patient has shown improvement in GCS. Histopathology confirmed it as hemorrhagic VS. Post-operatively, he had right HB (House- Brackemann) grade IV facial weakness which could not be appreciated in preoperative phase. He gradually recovered well and was able to walk without support at the time of discharge.

**Result:**

At 4 months follow up, facial weakness slightly improved to HB grade III, and patient was doing his daily activity without difficulty. Follow-up magnetic resonance imaging (MRI) imaging showed a small residue near internal acoustic meatus (IAM).

**Discussion and conclusion:**

Hemorrhage in VS is associated with increased morbidity and mortality; and few times, urgent intervention can save life despite of comatose state of the patient.

## Highlights

• Vestibular schwannoma with bleed is a rare phenomenon.
• These tumors becomes very challenging to manage because of significant increase in size causing brainstem compression and herniation.
• Urgent Cerebrospinal fluid (CSF) diversion in patients with Glasgow coma scale (GCS) score-3 can also revive the patient if attempted early.

## 1 Introduction

Vestibular schwannomas (VS) are benign neoplasm of cranial nerve (CN) VIII and arise from oligodendrocyte-schwann cell junction (Obersteiner-Redlich zone) and have incidence of 8–10% of all intracranial tumors (Tos et al., 2004). The incidence of giant VS (VS; ≥ 4 cm in size) in western countries is around 2% of all VS but in the Indian subcontinent, the incidence is still high and constitute approximately 40–50% of all VS (Jain et al., 2005). These tumors are slow-growing with growth rate of 1–2 mm/year, and with nonspecific features of headache and later developed cranial nerve deficits and cerebellar features (Niknafs et al., 2014).

Acute neurological deterioration due to gross intratumoral hemorrhage is exceedingly rare and occurs in less than 1% of cases (Carlson et al., 2010). Till now, 97 cases of VS with clinically significant hemorrhage have been reported in the literature but only 13 cases have giant VS out of them. We are presenting this unique case in view of giant VS with hemorrhage presented with poor neurological status (GCS-3 and mid dilated pupil) and we emphasized on the urgent intervention leading to functional recovery of the patient. Our article also stressed on the established histopathological features and also discussed the future pathological markers explaining the hemorrhagic nature of VS.

## 2 Case illustration

**Fig. 1:**
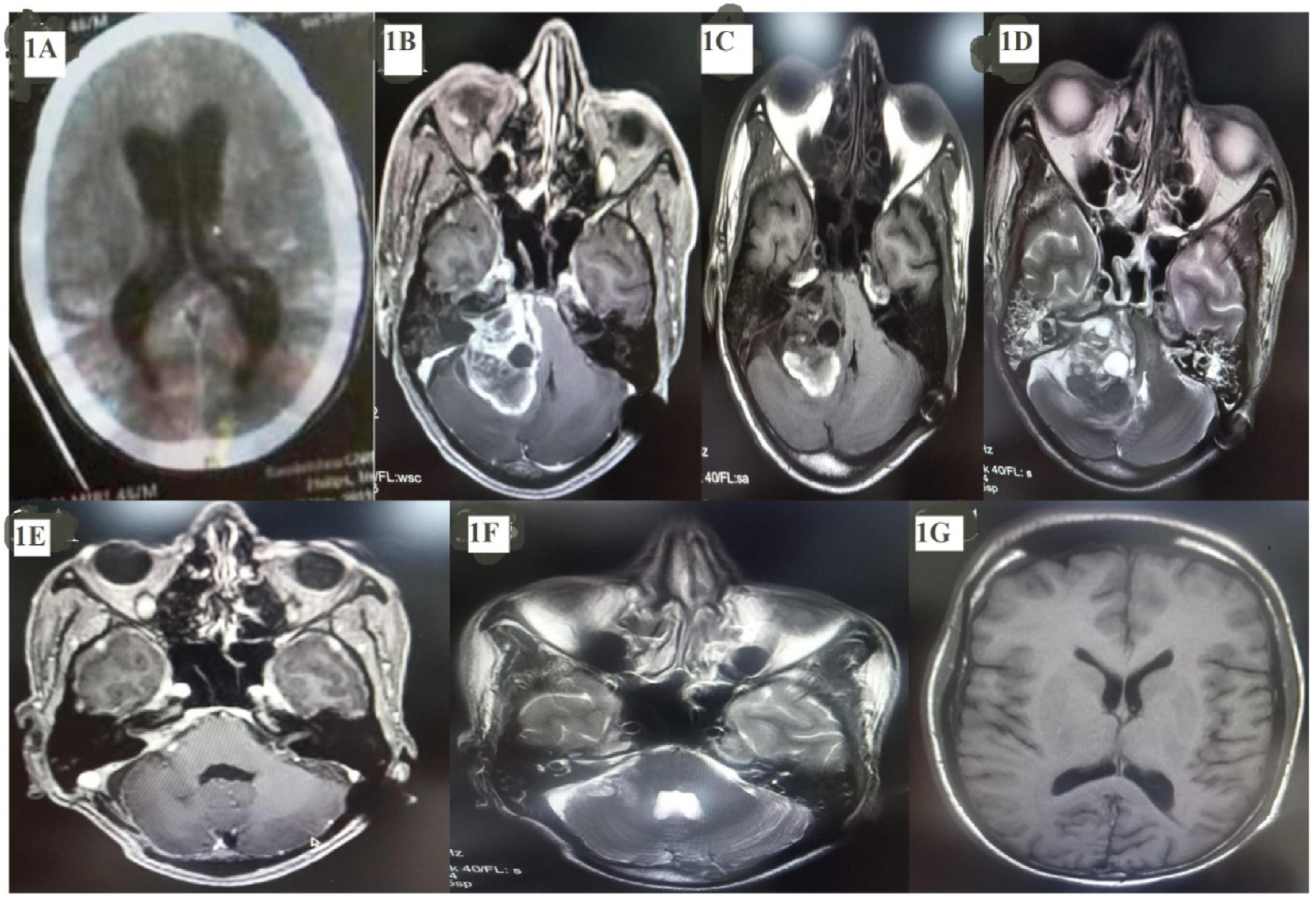
(1A) Computed tomography (CT) brain plain showed dilation of bilateral lateral ventricle suggestive of hydrocephalus at the time of admission. Fig. 1**: (1B)** T1 weighted with contrast MRI (Magnetic Resonance Imaging) showed 5.4 cm ​× ​3.8 cm sized enhancing solid tumor in right CP angle with extension into the right IAM. **(1C)** T1 weighted MRI image showed a predominant hypointense lesion in the right cp angle region extending into IAM, with hyperintensity in the periphery of lesion with severe brain stem compression. **(1D)** T2 weighted image revealed a hyperintense lesion in the right CP angle with hypointensity in the periphery of lesion suggestive of an early subacute phase of blood with no brainstem signal changes. **Figure 1(E,F,G) showed postoperative MRI- (1E)** T1 weighted with Gadolinium contrast MRI showed small residue in right IAM, in (1F) T2 weighted image showed postoperative changes seen in the right cerebellum and no brain stem compression. **(1G)** showed resolution of hydrocephalus.

A 42-year-old previously healthy man presented in the emergency room (ER), with a history of sudden onset severe headache, vomiting, followed by loss of consciousness with no history of previous similar episodes. On neurological examination, GCS was E1V1M1 and pupil bilateral mid-dilated, fixed. After immediate resuscitation including the intubation, Computed tomography (CT) brain was done. The imaging revealed a large hematoma with lesion in the right CP angle region, compressing the brain stem along with hydrocephalus (Fig. 1A). Urgent external ventricular drain (EVD) was placed in the ER and his sensorium was improved to GCS- E2VTM3 in next 6 hours. He was shifted to neurosurgery intensive care unit (NICU) for the mechanical ventilation; and EVD was converted into ventriculo-peritoneal (VP) shuntafter hemodynamic stabilization. Hearing and facial nerve assessment could not be possible due to poor neurological status. The patient was evaluated further with Magnetic Resonance Imaging (MRI) brain (plain and contrast) and T1 weighted image showed a predominant hypointense lesion in right CP angle region extending into internal acoustic meatus (IAM), with hyperintensity in the periphery, while T2 weighted image revealed central hyperintense lesion with hypointensity in the periphery suggestive of an early subacute phase of blood with significant brainstem compression. T1 weighted with gadolinium (Gd) contrast imaging revealed 5.4 ​× ​3.8 cm enhancing solid content with multicystic component of tumor in right CP angle with extension into the right IAM (Fig. 1B–D).

Under general anesthesia, in park bench position, patient underwent right retromastoid suboccipital craniotomy and excision of the lesion under cranial nerve neuro-monitoring. After craniotomy and dural opening, cerebellum was tense and bulging. On opening of the tumor capsule, hemorrhagic fluid under pressure came out. The lesion was soft to firm in consistency with multiple blood clots inside the lesion with good plain of cleavage. Near-total (>95%) excision was done and small part of the tumor capsule, densely adherent to the facial nerve near IAM was left behind. The lower cranial and facial nerves were identified and preserved. At the end of the surgery, the cerebellum was lax and pulsatile. The procedure was uneventful and patient was shifted to NICU for the elective ventilation. On the first postoperative day, he became conscious and obeying command but ventilator support was required for airway support. EVD was removed after 48 hours of surgery. He underwent tracheostomy on postoperative day (POD) 4, which was gradually closed on POD 15 after the partial recovery of lower cranial nerve palsy. During the postoperative phase, lateral tarsorrhaphy was also done in view of inadequate eye closure [House-Brackmann (HB grade IV)]. Histopathological examination was consistent with hemorrhagic VS. At the time of discharge, he had complete right hearing loss with right HB grade IV facial weakness and was able to walk without support.

At 4 months follow up, MRI brain revealed small residue near right IAM (Fig. 1E–G). On clinical examination, facial weakness improved to HB grade III, no auditory click noted in the right ear and he was doing his daily activity without difficulty.

## 3 Discussion

**Historical background-** Clinically significant hemorrhage in VS is a very rare occurrence in neurosurgical practice and requires urgent intervention. The incidence of acute neurological deterioration secondary to intratumoral hemorrhage (ITH) in VS is less than 1% (Carlson et al., 2010). The first case of intra-tumoral hemorrhage (ITH) in VS was reported by Mc Coyd et al., in 1974 (McCoYD and Cassidy, 1974). Till now, only 13 cases of giant VS (≥4 cm in size) with ITH have been reported (Table 1). Out of 13 previously reported cases of giant VS with ITH, only three cases presented with acute hydrocephalus and were managed with EVD or shunt (McCoYD and Cassidy, 1974; Carlson et al., 2010). Our case was unique as he was presented with very poor neurological status (GCS- E1M1V1) and in previous literature; no such case was reported with similar poor neurological status.

**Table 1:** Illustration of previous cases for giant VS with hemorrhage and their outcome.

Author/Year	Age/Sex	Presentation	Pre exiting diagnosis	Risk factor	Types of hemorrhage	Size(cm)	Facial weakness Pre op/post op	Follow up (months)/Outcome
Yonemitsu et al., 1983	49/M	HL, HA, Tinnitus	YES(6yr)	No	SDH, Intra tumoral	5	NA	24/good
Sasaki et al., 1985	33/F	HA, HL, tinnitus	NA	No	Intratumoral	4	NA	NR/Good
Ko et al., 1989	42/M	HA, HL, vomiting	Yes	No	Intratumoral	5	NA	NR/Good
Lee and Wang 1989	65/F	Ataxia, dizziness, HA, V	Yes	No	Intratumoral	4	NA	NR/Good
Kurata et al., 1989	56/M	HA, HL, vomiting	Yes	No	Intratumoral	5	NA	NR/Good
Palaoglu et al., 1990	41/M	Ataxia, HA, HL	NA	NO	Intratumoral	5.4	NA	NR/Good
Kim et al., 1998	35/F	HA, HL, Tinnitus	Yes	No	Intratumoral	4	NA/II	72/good
Dehdashti et al., 2009	26/F	HL, HA, V, vertigo	No	No	Intratumoral	4	II	NR/Good
Mathkour et al., 2018	40/M	HA, HL, Facial hypoesthesia	No	No	Intratumoral	4.1	II /II	12/Good
Yang et al., 2018	62/F	HA, HL, V, AT, Facial paresis	No	No	SAH, Intratumoral	4.1	III /II	24/Good
	56/M	HL,HA,DV, Facial paresis	No	No	SAH, Intratumoral	5.1	III /II	22/Good
	71/F	HL, Dizziness	Yes	HTN	Intratumoral	4.3	II /III	13/Good
	58/F	HA,V,HL,LOC	No	No	Intratumoral	5.1	III /II	8/Good
Present case	42/m	Sudden onset HA,LOC	NO	No	SDH, Intratumoral	5.4	CNBT /IV	4/Good

HA= Headache, HL= Hearing loss, V=Vomiting, AT ​= ​Ataxia, DV ​= ​double vision, SAH= Subarachnoid hemorrhage, LOC ​= ​Loss of consciousness, VP= Ventriculo-peritoneal, HB= House-Brackmann, SAH= Subarachnoid hemorrhage, SDH ​= ​subdural Hemorrhage, NR= Not Reported, CNBT ​= ​could not be tested.

**Risk factors and etiopathogensis for hemorrhage in Vestibular schwannoma-** Possible risk factors for hemorrhage in VS may be anticoagulants uses, trauma, pregnancy, and tumor size >2.5 cm (Niknafs et al., 2014). The possible mechanism of hemorrhage in such large lesion is due to the rapid growth of tumor, resulting in outgrowing of its blood supply and ultimately leading to necrosis, cystic degeneration, and hemorrhage (Niknafs et al., 2014; Ohta et al., 1998). Other factors as microvascular density, suggesting angiogenesis, have also shown its relationship to the tumor growth. It plays a vital role in the tumor growth and hypervascularity of the tumor, leading to recurrent microhemorrhages. Macroscopically, this hypervascularity may be explained by the dilated sinusoidal vessels with thin-walled and hyaline degeneration (Carlson et al., 2010, Niknafs et al., 2014). The pathological association with these abnormal vasculature findings has been credited to the Antoni b type pattern and explained the specific features of degeneration and the schwannoma's abnormal bleed vessels. Factors as increased matrix metalloproteinase II activity within the tumor also augment the vascular fragility and microhemorrhage, leading to cystic degeneration of VS. The tumor proliferation index estimated by the cell cycle markers as Ki-67 index and Histone H3 has been studied to correlate it with hemorrhagic nature of VS and found that they predict the change is growth pattern but it's role in hemorrhage is yet not established. Another detrimental factor studied was inflammation, assessed by CD 68 and CD 45 markers. It has been postulated that the inflammation also has a key role in causing hemorrhage in VS. From the previous literature, we can finally hypothesized that tumor growth, hypervascularity, and inflammation are the detrimental factors for hemorrhage in VS.

**Anticipated Cranial nerve deficits-** The reported incidence of CN VII palsy in all cases of VS is approximately 6%; whereas for the hemorrhagic VS, incidence of CN VII palsy is around 31.3% (Niknafs et al., 2014). The incidence of VII CN palsy is increased in hemorrhagic VS due to rapid tumor expansion, causing acute stretching of already splayed nerve over the tumor capsule (Niknafs et al., 2014). Certain studies including a large retrospective review of 773 patients with VS has shown that those with cystic VS were more likely to have higher HB grade facial nerve weakness.

**Mortality-** The mortality rate associated with VS is approximately 0.2%, whereas mortality rate reported in hemorrhagic VS is around 10% (Niknafs et al., 2014). With the advancement in microsurgical technique, mortality is no longer a major concern in the management of VS but timely intervention is decisive for good outcome in giant VS with ITH.

## 4 Conclusions

In conclusion, sudden spontaneous massive hemorrhage in VS is very rare but possible complication of VS. ITH may be provoked by anticoagulation status, trauma, rapid growth or previous radiation but in small subset of patients, hemorrhage may occur without any identifiable cause. Early microsurgical resection with clot evacuation in VS with ITH should be preferred management strategy for survival and better surgical outcome. The importance of pathological factors is of prime importance to study the pathophysiology of hemorrhage in vestibular schwannoma, which is a rare phenomenon in them.

## Declaration of competing interest

The authors declare that they have no known competing financial interests or personal relationships that could have appeared to influence the work reported in this paper.
